# Double chamber right ventricle in Williams syndrome: a rare cardiac anomaly reported

**DOI:** 10.1186/s40064-016-1897-y

**Published:** 2016-03-04

**Authors:** Jayitri Mazumdar, Rakesh Sarkar, Anusha Badveli, Biswajit Majumder

**Affiliations:** Department of Pediatrics, Calcutta National Medical College and Hospital, 24, Gorachand Road, Kolkata, 700014 India; R G Kar Medical College and Hospital, 1 Khudiram Bose Sarani, Kolkata, 700004 India

**Keywords:** Williams syndrome, Double chamber right ventricle, Bladder diverticula

## Abstract

Cardiovascular abnormality is the most consistent finding and occur in almost 80 % of all Williams syndrome (WS). Although a number of cardiovascular defects are common to WS, the majority presents in some form of arterial stenosis whereas supravalvular aortic stenosis is the most common one. Here we describe a 12 year old boy with elfin facies, presenting with urinary incontinence and a systolic murmur in right upper parasternal region. Echocardiography showed presence of double chambered right ventricle (DCRV) along with supravalvular aortic stenosis (SVAS) and coronary artery aneurysms, left pulmonary artery stenosis and multiple bladder diverticula in CT abdomen. With the clinical suspicion the diagnosis of WS was made and confirmed by fluorescent in situ hybridisation (FISH) study showing deletion in 7q11.23. Though different forms of arterial stenosis at multiple sites have been demonstrated in WS, DCRV in Williams syndrome is not reported till date in medical literature.

## Background

Williams syndrome constitutes of typical elfin facies, mental retardation, idiopathic hypercalcemia of infancy and supravalvular aortic stenosis (SVAS). Additional features include hypersensitivity to sound, spasticity, hypoplastic nails, dental anomalies, joint hypermobility, nephrocalcinosis, hypothyroidism, and poor weight gain. Williams syndrome has been shown to be due to a deletion involving the elastin gene on chromosome 7q11.23 (Pober 2010; Collins et al. 2010a, b; Bernstein 2015). The deletion 7q11.23 involves 26–28 genes including the *ELN* gene which codes the protein elastin (Wang et al. 1999). Hemizygosity of the *ELN* gene coding elastin has been demonstrated to be responsible for the vascular pathology in WS (Keating 1995).

In DCRV, a muscular band is present in the mid-right ventricular region, the band divides the chamber into two parts and creates obstruction between the inlet and outlet portions. The diagnosis of double-chambered right ventricle, which is commonly associated with ventricular septum defect, is usually made by echocardiography. The prognosis of the untreated cases of DCRV with right ventricular outflow tract (RVOT) obstruction at sub-infundibular level is similar to that for valvular pulmonary stenosis. When the obstruction is moderate to severe, surgery is indicated (Bernstein 2015).

The most common cardiac pathology in Williams is SVAS which is often associated with other defects like pulmonary stenosis and coronary artery abnormalities (Bernstein 2015). Here we describe the presence of DCRV, an unusual cardiac anomaly in a case of Williams syndrome along with other typical cardiac structural defects.

## The case

A 12 year male, the product of a non-consanguineous marriage presented with complaints of fever for last one-month and history of urinary incontinence. There was no history of joint pain, rash, convulsion or hematuria. The antenatal period was uneventful. Past history revealed that the patient had been suffering from urinary incontinence since his early childhood and parents could identify it at the age of 5 years by which the patient should have achieved bladder control.

**Fig. 1:**
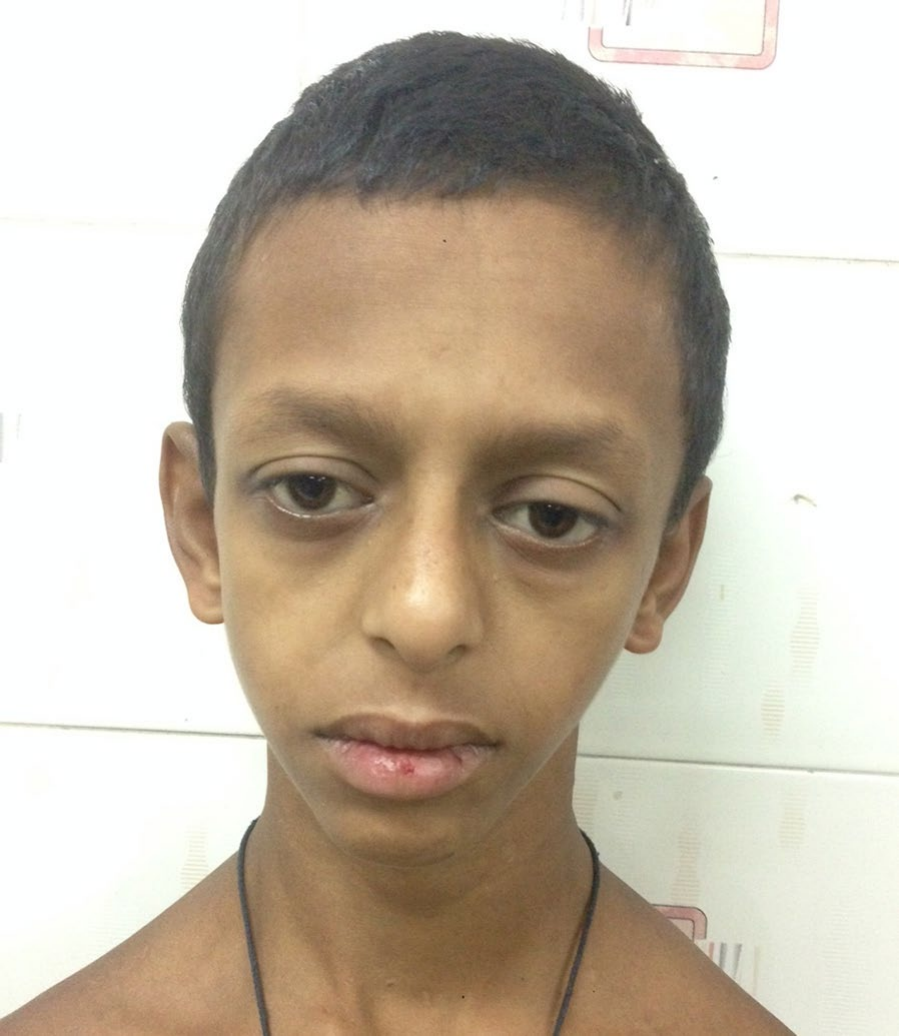
Showing typical elfin facies

On examination, he was found to have typical elfin facies (Fig. 1) with broad and thin upper lip, prominent ears, broad forehead, periorbital puffiness, flat nasal bridge (patient’s parents gave full consent to use the photograph and the medical details of their son).

On routine examination, cardiovascular system revealed an ejection systolic murmur of grade V/VI with systolic thrill in right upper sternal border at second intercostal space with radiation to both the carotid artery and another Ejection Systolic Murmur in the left second and third intercostal space of grade IV/VI. The patient had moderate degree of mental retardation with an IQ of 52.

Lab investigations showed that complete blood count, electrolytes, liver function test, urea, creatinine all were within normal limit. Serum calcium (corrected) was normal. Urine culture revealed presence of *E. Coli* >10^5^/ml.

**Fig. 2:**
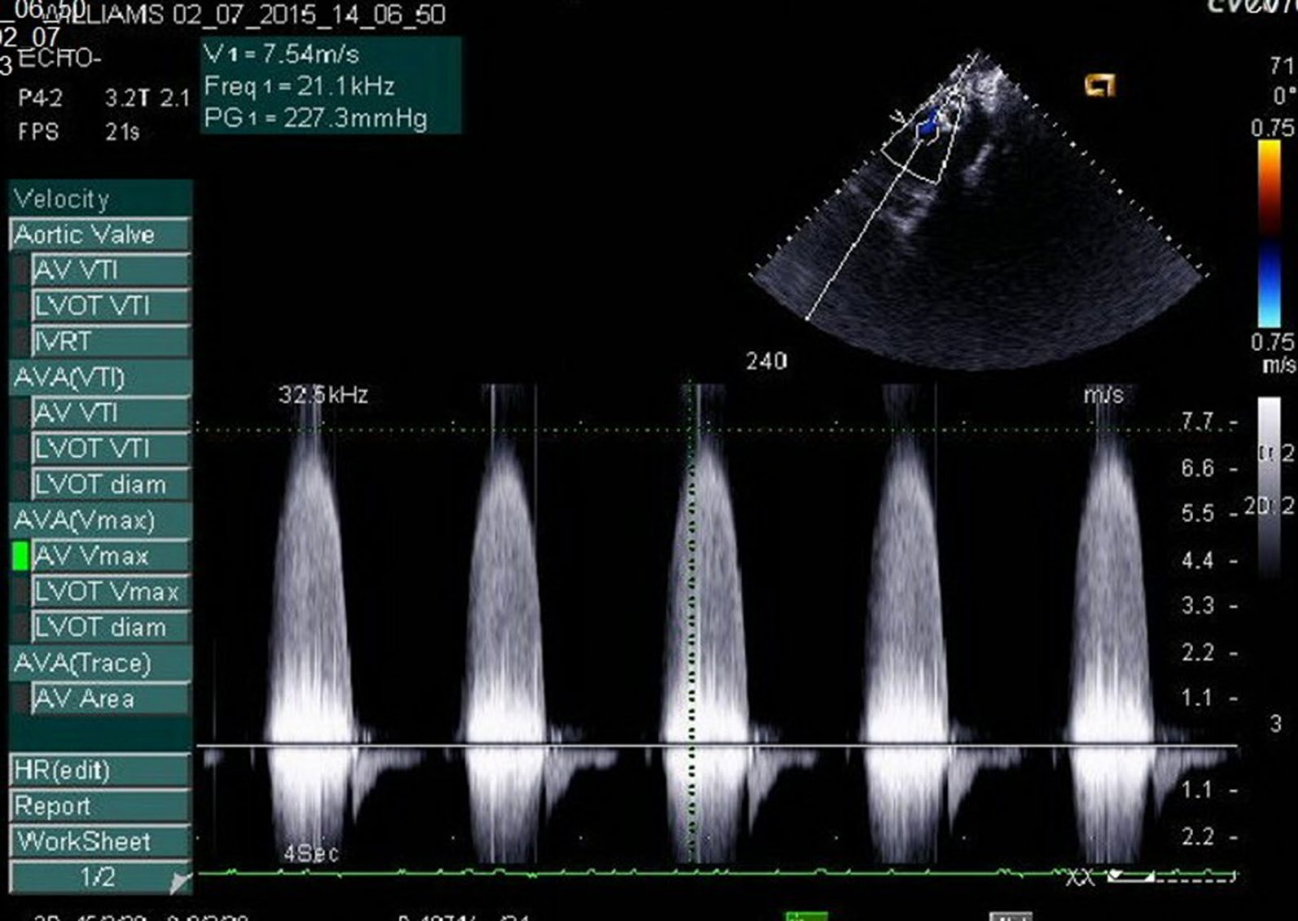
Continuous wave Doppler echocardiography in suprasternal view showing supravalvular aortic stenosis with peak systolic gradient of 227.3 mmHg

**Fig. 3:**
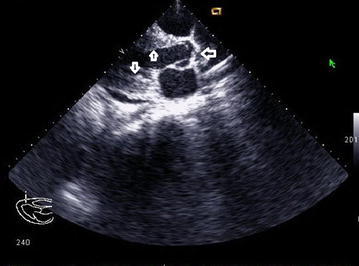
Echocardiography in parasternal long axis view showing supra-valvular aortic membrane causing SVAS in systolic phase (*horizontal arrowhead*) and increased left ventricular wall thickness (*vertical arrowhead*)

**Fig. 4:**
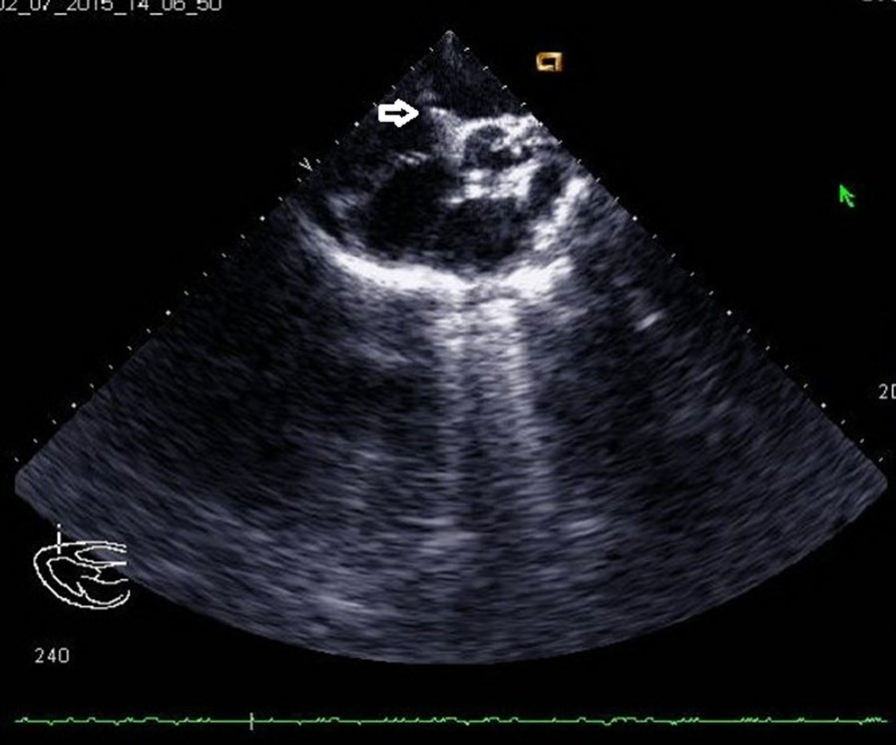
2Dechocardiography parasternal short axis view showing double chambered right ventricle (DCRV), *white arrowhead* showing the band dividing the ventricular cavity

**Fig. 5:**
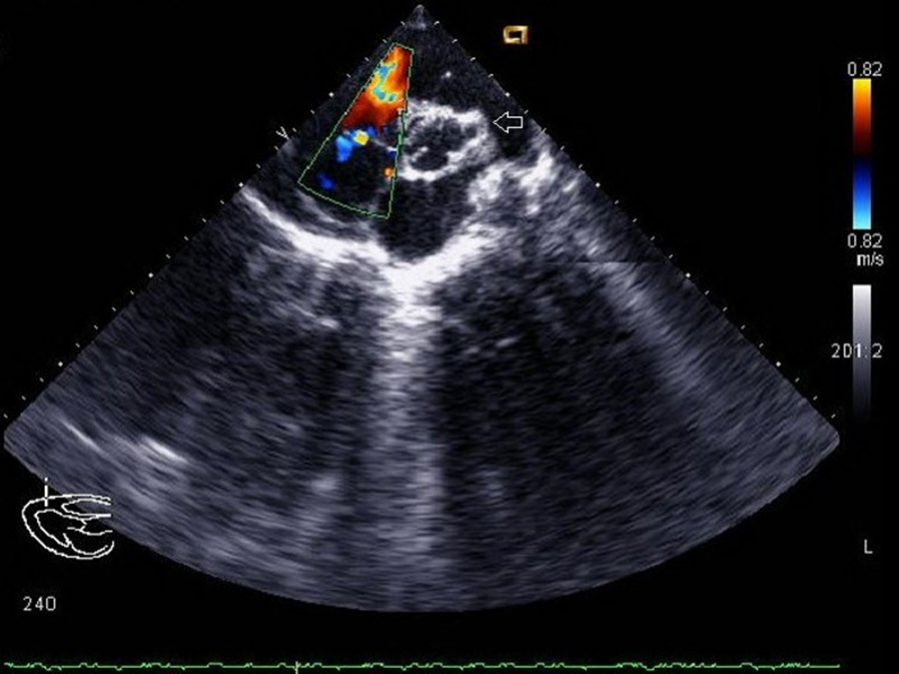
Color Doppler echocardiography of parasternal short axis view with leftward swept showing turbulent flow across the DCRV and mild tricuspid regurgitation along with left coronary artery aneurysm (*white arrowhead*)

2D echocardiography with Doppler study revealed a severe supravalvular aortic stenosis (peak gradient 227.3 mm of Hg) along with the presence of a supravalvular aortic membrane (Figs. 2 and 3) and severe left pulmonary artery stenosis of peak gradient 90 mmHg, concentric Left Ventricular Hypertrophy(LVH), a band like structure in the right ventricular cavity producing significant subinfundibular (intra-cavitary) RVOT obstruction with peak systolic gradient of 68 mmHg acting as functionally double chambered right ventricle (DCRV) (Figs. 4 and 5). Echocardiography also revealed aneurysmally dilated left main coronary artery having a diameter of 8.9 mm before the bifurcation left anterior descending and left circumflex (Fig. 5). But there was no evidence of VSD, not even partially closed.

Ultrasonography (USG) and contrast enhanced computed tomography (CECT) of abdomen revealed multiple bladder diverticula with bilateral hydronephrosis. After initial treatment with appropriate intravenous antibiotics as per urine culture sensitivity, the patient was planned for genetic evaluation.

**Fig. 6:**
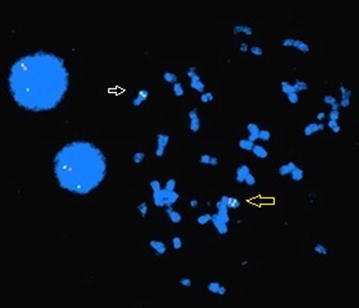
FISH study using a unique sequenced probe that hybridizes to the elastin gene on Chromosome 7q11.23. The elastin probe is labeled in *red* and the control probe on chr. 7 is labeled in *green*. *Large arrowhead* shows a normal chromosome with both *red* and *green* signals for elastin region and control probe, whereas *small arrowhead* shows only two green signals for the control probes which corresponds to 7q11.23 deletion

Fluorescent in situ hybridization (FISH) was performed, and the result showed del (7q11.23) compatible with Williams’ syndrome (Fig. 6).

Henceforth the patient was referred to cardiothoracic department for surgical correction. Advice for corrective surgery was given but it was delayed due to financial constraints faced by the patient’s relative. Though the patient was initially hemodynamically stable with no signs of cardiac decompensation, the patient developed chest heaviness and shortness of breath on follow up within 2 months of discharge. The patient had been readmitted due to these cardiac symptoms and finally died prior to surgery.

## Discussion

Supravalvular aortic stenosis (SVAS) is the cardiovascular lesion first reported by Williams et al. and has been found to be the most common cardiovascular abnormality in Williams Syndrome (WS) (Pober 2010; Collins et al. 2010a). The incidence of SVAS has been reported to be present among 45–75 % patients with WS. Arterial narrowing may be isolated or may be seen simultaneously in multiple locations, including the aortic arch, the descending aorta, and the pulmonary, coronary, renal, mesenteric, and intracranial arteries. Among the other cardiac manifestations, pulmonary stenosis, ventricular and atrial septal defect are also seen in Williams syndrome Collins et al. (2010b). Patients with WS and with hemizygosity of *ELN* lack the elasticity of the arterial wall provided by normal elastin and thereby have increased arterial stiffness (Wang et al. 1999; Keating 1995).

In DCRV, a muscular band which is present in the mid-right ventricular region, divides the chamber into two parts and creates obstruction between the inlet and outlet portions. An associated VSD that may close spontaneously is often noted. Obstruction is not usually seen early in life in DCRV but may progress rapidly in a similar manner to the progressive infundibular obstruction observed with tetralogy of Fallot (Bernstein 2015). Occurrence of DCRV in WS is extremely rare and not reported in medical literature till date.

The Most unusual thing about our case is that the patient had no complaints of dyspnoea, palpitation, chest pain initially. He presented only with urinary incontinence and CT abdomen revealed multiple bladder diverticula, which sometimes may be associated with Williams syndrome (Babbitt et al. 1979). But it was the typical facies and heart murmur that dragged us for echocardiography revealing coronary artery anomalies along with supravalvular aortic stenosis, left pulmonary artery stenosis and most surprisingly DCRV, not reported till date in medical literature in association with WS. Finally WS was confirmed by FISH study.

## Conclusion

Though our patient presented initially with urinary incontinence, one of the non-cardiac symptoms, on routine examination, the elfin facies, neurocognitive deficit and the cardiovascular abnormality detected by Echocardiography led to the diagnosis of Williams syndrome which was also supported by genetic analysis. However the patient ultimately succumbed to some cardiovascular complications. We here suggest for paying much attention on complete clinical examination while evaluating any symptom especially in a patient with dysmorphic facies, as sometimes that can explore a rare syndrome which may have even much fatal component underneath.
